# Scutellarin attenuates hypoxia-induced retinal neovascularization through bidirectional modulation of the VEGF/Ang/Tie2 pathway

**DOI:** 10.3389/fphar.2025.1631316

**Published:** 2025-10-01

**Authors:** Shan Ding, Wei Wang, Chunmeng Liu, Wenfeng Zhang, Jie Wang, Fuwen Zhang

**Affiliations:** ^1^ Eye School of Chengdu University of Traditional Chinese Medicine, Chengdu, Sichuan, China; ^2^ Ineye Hospital of Chengdu University of Traditional Chinese Medicine, Chengdu, Sichuan, China; ^3^ Key Laboratory of Sichuan Province Ophthalmopathy Prevention & Cure and Visual Function Protection with TCM Laboratory, Chengdu, Sichuan, China

**Keywords:** scutellarin, retinal neovascularization, VEGF/Ang/Tie2 signaling pathway, bidirectional regulation, faricimab, hypoxia

## Abstract

**Introduction:**

Scutellarin (SCU), a flavonoid with established pharmacological activity, exhibits anti-angiogenic and vascular-stabilizing effects in ischemic ocular diseases.

**Methods:**

This study investigated its bidirectional modulation of the vascular endothelial growth factor(VEGF)/Angiopoietin (Ang)/Tie2 signaling pathway in retinal neovascularization (RNV) under hypoxic conditions and compared its efficacy with Faricimab, a dual-target angiogenesis inhibitor. A CoCl_2_-induced hypoxic model in rat retinal microvascular endothelial cells (rRMECs) was used to evaluate proliferation, migration, and tube formation. Network pharmacology and molecular docking were employed to predict SCU targets and key signaling pathways. Western blotting and qRT-PCR validated its regulatory effects at the molecular level.

**Results:**

SCU significantly and dose-dependently suppressed rRMEC proliferation, migration, and tube formation under hypoxia, with effects comparable to those of Faricimab at higher concentrations. Network pharmacology identified 43 overlapping targets between SCU and RNV. Pathway enrichment analysis indicated involvement of VEGF, MAPK, and Ras signaling. Molecular docking showed strong binding of SCU to VEGF-A, Ang2, Tie2, and Vascular endothelial protein tyrosine phosphatase (VE-PTP). SCU downregulated pro-angiogenic factors (VEGF-A, Ang2, HIF-1α, VE-PTP) and upregulated vascular stability-related proteins (Ang1, Tie2, vascular endothelial cadherin (VE-cadherin)) at both mRNA and protein levels.

**Discussion:**

These results suggest that SCU exerts a dual regulatory effect on retinal neovascularization by simultaneously inhibiting pathological angiogenesis and enhancing vascular stabilization via the VEGF/Ang/Tie2 signaling pathway. Its mode of action complements and extends the mechanism of Faricimab, supporting its potential as a promising natural candidate for retinal vascular disease therapy.

## 1 Introduction

Retinal neovascularization (RNV) is a common pathological changes of several intractable retinal diseases, including proliferative diabetic retinopathy (PDR), neovascular age-related macular degeneration (nAMD), retinal vein occlusion, and retinopathy of prematurity (Gau et al., 2020; Ji et al., 2020). It is primarily induced by retinal ischemia, hypoxia, and microcirculatory dysfunction (Carmeliet and Jain, 2011), and is characterized by abnormal vessel proliferation, increased vascular permeability, and breakdown of the blood-retinal barrier, ultimately leading to irreversible visual impairment (Xu et al., 2022). Hypoxia is considered as the central trigger of RNV, promoting pathological angiogenesis via upregulation of HIF-1α and subsequent activation of vascular endothelial growth factor (VEGF) signaling (Apte et al., 2019). Although intravitreal injection of anti-VEGF agents remains the primary treatment for RNV, monotherapy targeting VEGF has limitations, including incomplete efficacy, drug resistance, and potential adverse events such as retinal detachment and further vision loss (Uemura et al., 2021).

Recently, the Angiopoietin (Ang)/Tie2 signaling pathway has emerged as a critical regulator of vascular stability. Ang1 activates the Tie2 receptor to promote endothelial cell junction integrity and vascular quiescence, while Ang2, upregulated under hypoxic conditions, antagonizes Ang1, disrupts Tie2 signaling, and increases vascular leakage and instability (Li et al., 2019). This highlights the therapeutic potential of dual modulation of the VEGF/Ang/Tie2 axis—simultaneously suppressing VEGF/Ang2 and activating Tie2—to achieve anti-angiogenesis and vascular stabilization. Faricimab, a bispecific monoclonal antibody targeting both VEGF-A and Ang2, has been approved for treating nAMD and diabetic macular edema (DME), (Koh et al., 2022), and demonstrates superior durability and safety compared with anti-VEGF monotherapy (Heier et al., 2022; Wykoff et al., 2022; Chen-Li et al., 2024). However, there is no direct evidence that Faricimab activates Tie2, and its precise mechanisms and long-term safety remain to be elucidated.

*Erigeron breviscapus* (Vaniot) Hand.-Mazz, a traditional Chinese herb known for its blood-activating and microcirculation-improving properties, has shown therapeutic potential in the treatment of RNV(Mei et al., 2019; Nie et al., 2024). Our research group developed a traditional Chinese medicine (TCM) formula named Qidengmingmu Capsule, composed of E. breviscapus, *Astragalus membranaceus* (Fisch.) Bunge, and *Pueraria montana* (Lour.) Merr. Scutellarin (SCU), a major flavonoid compound extracted from E. breviscapus (Wang et al., 2023), has been identified as one of the key active constituents of this formula. Modern pharmacological studies have shown that SCU exhibits antioxidant (Mo et al., 2018), anti-inflammatory (Chen et al., 2020), antitumor (Ma et al., 2024), and anti-angiogenic effects (Long et al., 2019). Meanwhile, SCU can alleviate microvascular dysfunction caused by hyperglycemia and hypoxia and suppress VEGF-mediated retinal neovascularization (Xie et al., 2024). Our previous research also demonstrated that SCU significantly inhibits RNV progression, though its precise mechanism of action remains unclear. Therefore, investigating whether SCU exerts a bidirectional regulatory effect on the VEGF/Ang/Tie2 pathway—namely, inhibiting angiogenesis while promoting vascular stability—has significant therapeutic implications for retinal vascular diseases.

**FIGURE 1:**
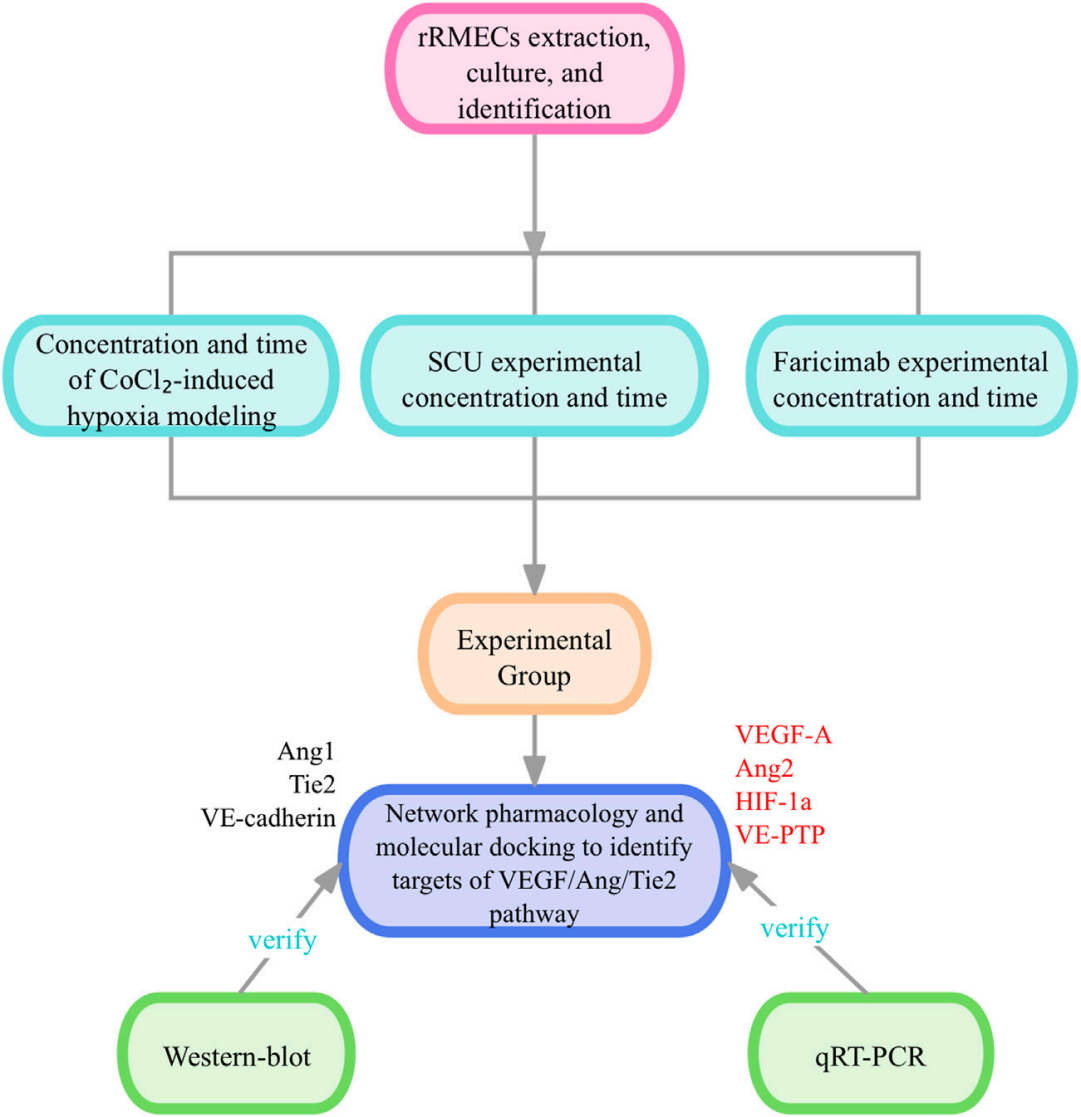
The flow chart of the experiment.

Therefore, this study aims to investigate whether SCU exerts a bidirectional regulatory effect on the VEGF/Ang/Tie2 pathway by inhibiting pathological angiogenesis while enhancing vascular stability. Using a CoCl_2_-induced hypoxic model in rat retinal microvascular endothelial cells (rRMECs), we evaluated SCU’s effects on RNV-related cellular behaviors, compared its efficacy with Faricimab, and explored its molecular targets via network pharmacology, molecular docking, and experimental validation (Figure 1).

All procedures were reviewed and approved by the Animal Ethics Committee of Chengdu University of Traditional Chinese Medicine (CDUTCM) (Approval No. 2019–30).

## 2 Materials and methods

### 2.1 Cells and reagents

rRMECs were isolated and cultured in-house. The following reagents were used in the study: Scutellarin (SCU) (Chengdu Aifa Biotechnology Co., Ltd., CAS No. 3681-99-0); CoCl_2_ and Type II collagenase (Sigma-Aldrich, United States, Catalog No. C8661 and C6885); Ang1 rabbit monoclonal antibody (Abclonal, China, Catalog No. A3757, dilution 1:1,000); Ang2 rabbit monoclonal antibody (Zenbio, China, Catalog No. 382015, dilution 1:1,000); VEGF-A rabbit monoclonal antibody (Abclonal, China, Catalog No. A0280, dilution 1:1,000); Tie2 rabbit monoclonal antibody (Proteintech, United States, Catalog No. 19157-1-AP, dilution 1:500); HIF-1α rabbit monoclonal antibody (Abclonal, China, Catalog No. A6265, dilution 1:1,000); VE-PTP rabbit monoclonal antibody (Thermo Fisher Scientific, United States, Catalog No. PA5-104088, dilution 1:500); VE-cadherin rabbit monoclonal antibody (Abclonal, China, Catalog No. A0734, dilution 1:1,000); CCK-8 (APExBIO, United States, Catalog No. K1018); Endothelial cell medium (ECM) (ScienceCell, United States, Catalog No. 1001); Fibronectin (FN) (Beomei Biotechnology, Hefei, China, Catalog No. XF0531); ABW Gold Matrigel (Nova Biotechnology, Shanghai, China, Catalog No. 0827045); von Willebrand factor antibody (vWF) (Thermo Fisher Scientific, United States, Catalog No. PA5-16634); Penicillin-Streptomycin solution (Hyclone, United States, Catalog No. SV30010); Phosphate-buffered saline (PBS) (Corning, United States, Catalog No. R1600); Fetal bovine serum (FBS) (Corning, United States, Catalog No. 35-076-CV); 4% paraformaldehyde (Solarbio, China, Catalog No. P1110); 0.1% Triton X-100 (Solarbio, China, Catalog No. T8200); Goat anti-rabbit IgG (HRP-conjugated) (Proteintech, China, Catalog No. SA00006-2); DAPI (Solarbio, China, Catalog No. C0060); Fluoromount-G mounting medium (SouthernBiotech, United States, Catalog No. 0100-01); 0.4% Trypan Blue staining solution (Pusainuo Biotechnology, Wuhan, China, Catalog No. PB180423).

### 2.2 Cell isolation, culture, and identification

#### 2.2.1 Isolation and culture of rRMECs

Six male specific-pathogen-free Sprague-Dawley rats (6–8 weeks old, 200 ± 20 g) were obtained from the Animal Experimental Center of CDUTCM (License No. SCXK [Chuan] 2019-11). After routine ocular disinfection, the eyeballs were enucleated, and retinas were dissected. Following PBS washing, the retinal tissues were cut into ∼1 mm^3^ fragments. The tissue was digested with 0.1% Type II collagenase at 37 °C for 15 min, filtered through a 300 μm nylon mesh, and centrifuged to remove the supernatant. The resulting cell pellet was resuspended in 6 mL of complete endothelial growth medium (containing 10% fetal bovine serum, 1% penicillin-streptomycin, and 1% endothelial cell growth supplement). The cell suspension was seeded into T25 culture flasks pre-coated with 50 μg of fibronectin and incubated at 37 °C in 5% CO_2_. Half of the medium was changed after 48 h, and complete medium was refreshed every 2–3 days thereafter. Cell adherence, morphology, density, and confluence were monitored using an inverted phase-contrast microscope. When the cell confluence reached 85%–90%, the cells were passaged. Third-to fifth-passage cells were used for subsequent experiments.

#### 2.2.2 Cell identification

The identity of the isolated rRMECs was confirmed by morphological assessment and immunofluorescent staining for vWF. Cells were seeded at a density of 2 × 10^4^ cells/well on sterilized coverslips in 24-well plates. After adherence, cells were fixed with 4% paraformaldehyde for 30 min and permeabilized using 0.1% Triton X-100. Non-specific binding was blocked with 5% goat serum for 1 h at room temperature. Cells were then incubated with a rabbit anti-mouse vWF polyclonal primary antibody (1:100) overnight at 4 °C, followed by a FITC-conjugated goat anti-rabbit IgG secondary antibody (1:500) for 2 h at room temperature in the dark. Nuclei were counterstained with DAPI (1:1,000) for 5 min. In the negative control group, PBS was used in place of the primary antibody. Coverslips were mounted with Fluoromount-G and observed under a fluorescence microscope.

### 2.3 Determination of optimal drug concentrations using CCK-8 assay

#### 2.3.1 Establishment of CoCl_2_-Induced hypoxia model

To determine the optimal hypoxia-inducing conditions, rRMECs were seeded in 96-well plates (1 × 10^5^ cells/mL) and incubated for 24 h. Except for the control group, the remaining groups were treated with CoCl_2_ at various concentrations (50, 100, 200, 300, 400 μmol/L) for 24 h or 48 h. CCK-8 reagent (10 μL) was then added to each well, followed by 2 h incubation at 37 °C. Absorbance at 450 nm was measured to calculate cell viability and determine the optimal concentration and duration of CoCl_2_ treatment.

#### 2.3.2 Determination of SCU treatment concentration

After rRMECs were seeded and adhered, they were divided into the following groups: control (ECM medium), vehicle control (0.1% DMSO), and SCU groups (1, 5, 10, 50, 100, 500 μmol/L). Cell viability was assessed 24 h after treatment using the CCK-8 assay to determine the non-toxic concentration range of SCU.

#### 2.3.3 Determination of faricimab treatment concentration

rRMECs were cultured and divided into a control group (ECM medium) and Faricimab treatment groups (2, 4, 20, 40, 200, 400, 2000 ng/mL), followed by 24 h incubation. Cell viability was evaluated using the CCK-8 assay to determine the optimal treatment concentration.

### 2.4 Cell proliferation assay

Fourth-passage rRMECs were seeded in 96-well plates (1 × 10^5^ cells/mL, 100 μL per well). After cell adherence, CoCl_2_ (200 μmol/L) was added for 24 h to induce hypoxia. Cells were then treated with SCU (5 or 50 μmol/L), Faricimab (400 ng/mL), or ECM medium (control) for an additional 24 h. OD values were measured at 450 nm using the CCK-8 assay to calculate proliferation rates.

### 2.5 Cell migration assays

#### 2.5.1 Wound healing assay

Five parallel lines were drawn on the bottom of each well of a 6-well plate. Cells were seeded and cultured for 24 h until confluence. A sterile 200 μL pipette tip was used to create three vertical scratches across the lines. After PBS washes, treatment agents were added. Images were captured at 24 h and 48 h, and migration area was quantified using ImageJ.

#### 2.5.2 Transwell migration assay

Cell migration was assessed using Transwell chambers with 8.0 μm pores. rRMECs (5 × 10^4^ cells/well) were seeded in the upper chamber with 200 μL serum-free medium, while 600 μL of medium with 10% FBS was added to the lower chamber. After 6 h, treatment was applied for 24 h. Cells were fixed with 4% paraformaldehyde, stained with 0.1% crystal violet, and non-migrated cells were removed. The number of migrated cells was counted in five randomly selected fields under an inverted microscope.

### 2.6 Tube formation assay

Matrigel was pre-coated onto 96-well plates. Hypoxia-induced rRMECs were seeded and treated for 8 h according to the experimental groups. Tube formation was observed under an inverted microscope. Images were taken from five random fields per well, and branches and junctions were quantified using ImageJ.

### 2.7 Network pharmacology and molecular docking

#### 2.7.1 Target identification of SCU and RNV

Putative SCU targets were predicted using PharmMapper (http://www.lilab-ecust.cn/pharmmapper) and Swiss Target Prediction (http://www.swisstargetprediction.ch). Target names were normalized via UniProt (http://www.uniprot.org). Potential RNV-related targets were obtained using the keyword “Retinal Neovascularization” from GeneCards (https://www.genecards.org). Common targets were identified using Venny 2.1.0.

#### 2.7.2 Protein–protein interaction (PPI) network construction

Following the method by Chen (Chen et al., 2024), common targets were imported into STRING (https://cn.string-db.org/) for PPI analysis. Visualization was performed using Cytoscape 3.9.1, and topological parameters (Degree) were calculated using NetworkAnalyzer. Core hub genes were identified with the CytoHubba plugin.

#### 2.7.3 GO and KEGG enrichment analysis

Following the method by Cao(Cao et al., 2024), enrichment analyses were performed using the ClusterProfiler package in Gene Ontology (GO) terms and Kyoto Encyclopedia of Genes and Genomes (KEGG) pathways with *p* < 0.05 and FDR < 0.05 were considered significant; the top 20 pathways were visualized.

#### 2.7.4 Molecular docking

SCU structure was obtained from PubChem and prepared using LigPrep in Maestro 12.5. PDB structures of Ang1, Ang2, Tie2, HIF-1α, VEGF-A, vascular endothelial-protein tyrosine phosphatase (VE-PTP), and Vascular endothelial cadherin (VE-cadherin) were retrieved from the RCSB Protein Data Bank. Proteins were pre-processed using the Protein Preparation Wizard (adding hydrogens, deleting water, optimizing geometry). Docking was performed using the XP mode. Binding energies (kcal/mol) were evaluated, with ≤ −5.0 kcal/mol indicating strong binding. Binding modes were visualized and analyzed for hydrogen bonds, π–π stacking, and electrostatic interactions using PyMOL and Discovery Studio.

### 2.8 Western-blot

rRMECs in log phase were grouped, treated accordingly, and lysed to extract total proteins. Protein concentrations were measured via BCA assay. SDS-PAGE was performed, followed by electrotransfer, blocking, primary and secondary antibody incubation. Bands were detected via chemiluminescence and quantified using Image Lab 3.0 with GAPDH as internal reference.

### 2.9 qRT-PCR

rRMECs were seeded in 10-cm dishes, treated according to the experimental design. Total RNA was extracted using a commercial kit, reverse transcribed to cDNA, and analyzed by qRT-PCR. GAPDH was used as internal control. Relative gene expression was calculated using the 2^−ΔΔCT^ method. All primers were synthesized by Sangon Biotech (Shanghai); sequences are listed in Table 1.

**TABLE 1:** q-RTPCR primers and base sequences.

Gene name	Primer sequences (Forward primer/Reverse primer)	Length/bp
GAPDH	ACGGCAAGTTCAACGGCACAG CGACATACTCAGCACCAGCATCAC	129
Ang1	GATGAGCCTGCGTCCTCTGTTG ATCTGGCATCCCGACCCTTGG	140
Ang2	AGCCAGTCTCCCTTCCAGATCAC GGACAGGCAAGCCATTCTCACAG	127
Tie2	GTTGAGAGGTGGTCCCAGCAAAC TTGTTCAGCAGCACGGAAGTCAG	83
HIF-1α	CCGCCACCACCACTGATGAATC TTGTTCAGCAGCACGGAAGTCAG	140
VEGF-A	CACGACAGAAGGGGAGCAGAAAG GGCACACAGGACGGCTTGAAG	150
VE-PTP	TACTTCGCCGTGGTGGTGAGAG GCGGATGGAGGCATTGTGTCTG	108
VE-cadherin	AAGAACGAGGACAGCAACTTCACC CAGGCAGGTAGTGGAACTTGGTATG	112

### 2.10 Statistical analysis

All statistical analyses were performed using SPSS version 26.0 (IBM Corp., Armonk, NY, United States). Quantitative data were expressed as mean ± standard deviation (mean ± SD). One-way analysis of variance (ANOVA) was used for comparison among multiple groups, followed by the least significant difference (LSD) test for pairwise comparisons. All experiments were independently performed at least three times. A P-value of <0.05 was considered statistically significant, while P < 0.01 was regarded as highly significant.

## 3 Results

### 3.1 Isolation, culture, and identification of rRMECs

**FIGURE 2:**
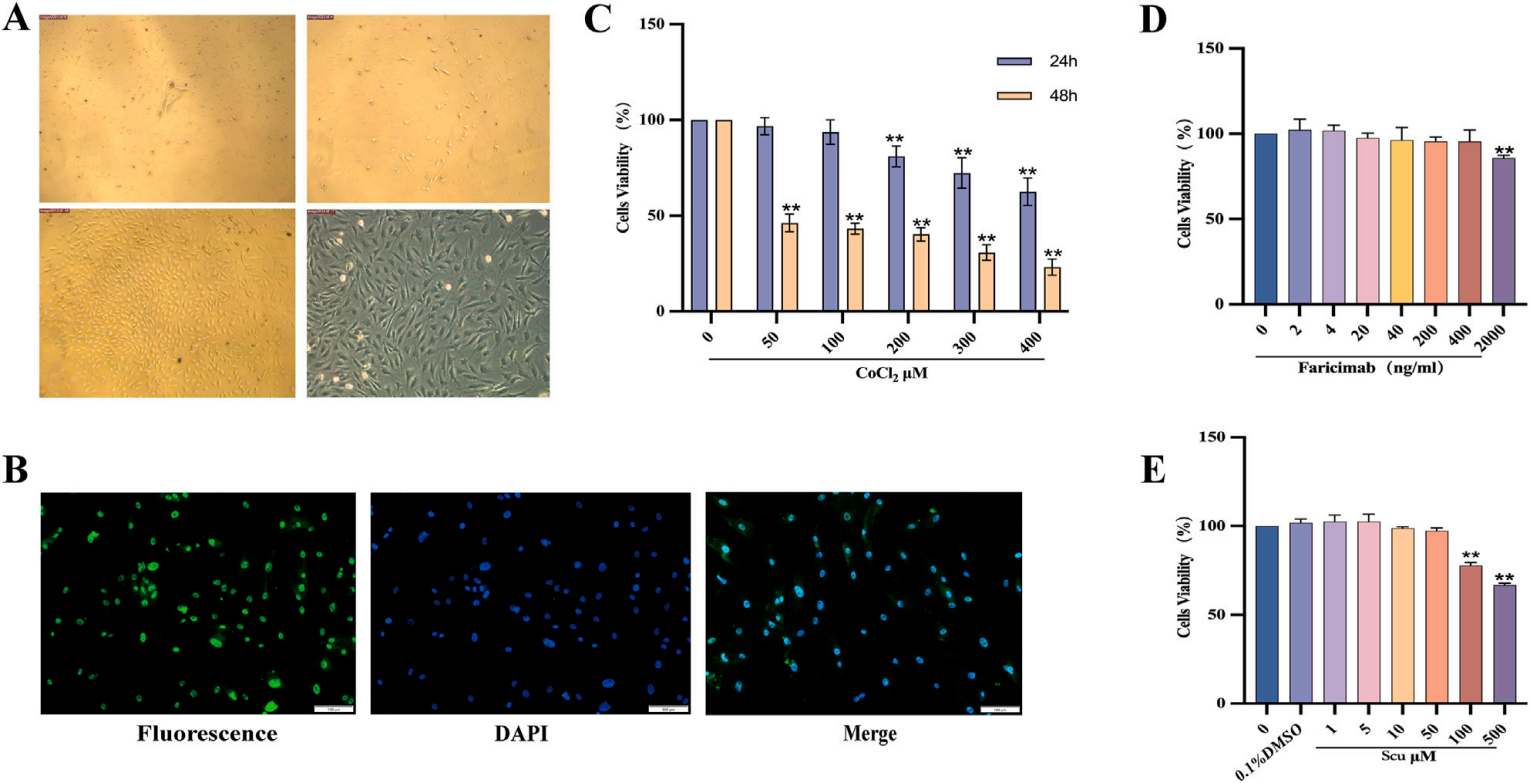
Identification of rRMECs, establishment of hypoxia model, and concentration screening of SCU and Faricimab. **(A)** Morphological observation of cultured rRMECs after 48 h. **(B)** Immunofluorescence staining of vWF showing positive cytoplasmic green fluorescence and DAPI-stained nuclei (×100). **(C)** Optimization of CoCl_2_ concentration and exposure time for hypoxic modeling. **(D)** Cell viability under various concentrations of Faricimab. **(E)** Cell viability under various concentrations of SCU. *P < 0.05, **P < 0.01 vs. Control group.

After 48 h of culture, rRMECs gradually migrated out from the retinal explants, displaying spindle-shaped, triangular, or polygonal morphologies. By days 7–10, the cells reached confluence, forming a cobblestone-like monolayer without apparent contact inhibition (Figure 2A). Immunofluorescence staining revealed strong positive expression of vWF in the cytoplasm (green fluorescence), with nuclei counterstained by DAPI (blue), and a positivity rate exceeding 90% (Figure 2B), confirming the identity of the cultured cells as rRMECs.

### 3.2 CCK-8 assay for cell viability

#### 3.2.1 Establishment of CoCl_2_-Induced hypoxia model

As shown in Figure 2C, increasing concentrations of CoCl_2_ progressively reduced rRMEC viability. Treatment with 200 μmol/L CoCl_2_ for 24 h significantly inhibited cell viability (*P* < 0.05) without causing complete cytotoxicity. In contrast, 48 h treatment with all tested concentrations resulted in a marked reduction in viability. Therefore, 200 μmol/L CoCl_2_ for 24 h was selected as the optimal hypoxia-inducing condition.

#### 3.2.2 Determination of SCU and faricimab concentrations

According to the CCK-8 assay (Figure 2D), 400 ng/mL Faricimab did not significantly reduce cell viability (*P* > 0.05) but exhibited effective anti-proliferative activity. SCU showed no cytotoxicity at concentrations between 1 and 50 μmol/L (Figure 2E). However, at concentrations ≥50 μmol/L, cell viability was significantly suppressed (*P* < 0.01). Thus, 5 μmol/L and 50 μmol/L were selected as the low and high dose groups, respectively. Additionally, 0.1% DMSO had no effect on viability (*P* > 0.05), confirming the absence of solvent interference.

#### 3.2.3 Experimental grouping

According to the results of preliminary CCK-8 screening, the following five experimental groups were used throughout subsequent assays: (1) Control; (2) Hypoxia (200 μmol/L CoCl_2_); (3) Faricimab (200 μmol/L CoCl_2_+400 ng/mL); (4) SCU-50 μmol/L (200 μmol/L CoCl_2_+ SCU-50 μmol/L); (5) SCU-5 μmol/L (200 μmol/L CoCl_2_+ SCU-50 μmol/L).

### 3.3 SCU inhibits hypoxia-induced rRMECs proliferation

**FIGURE 3:**
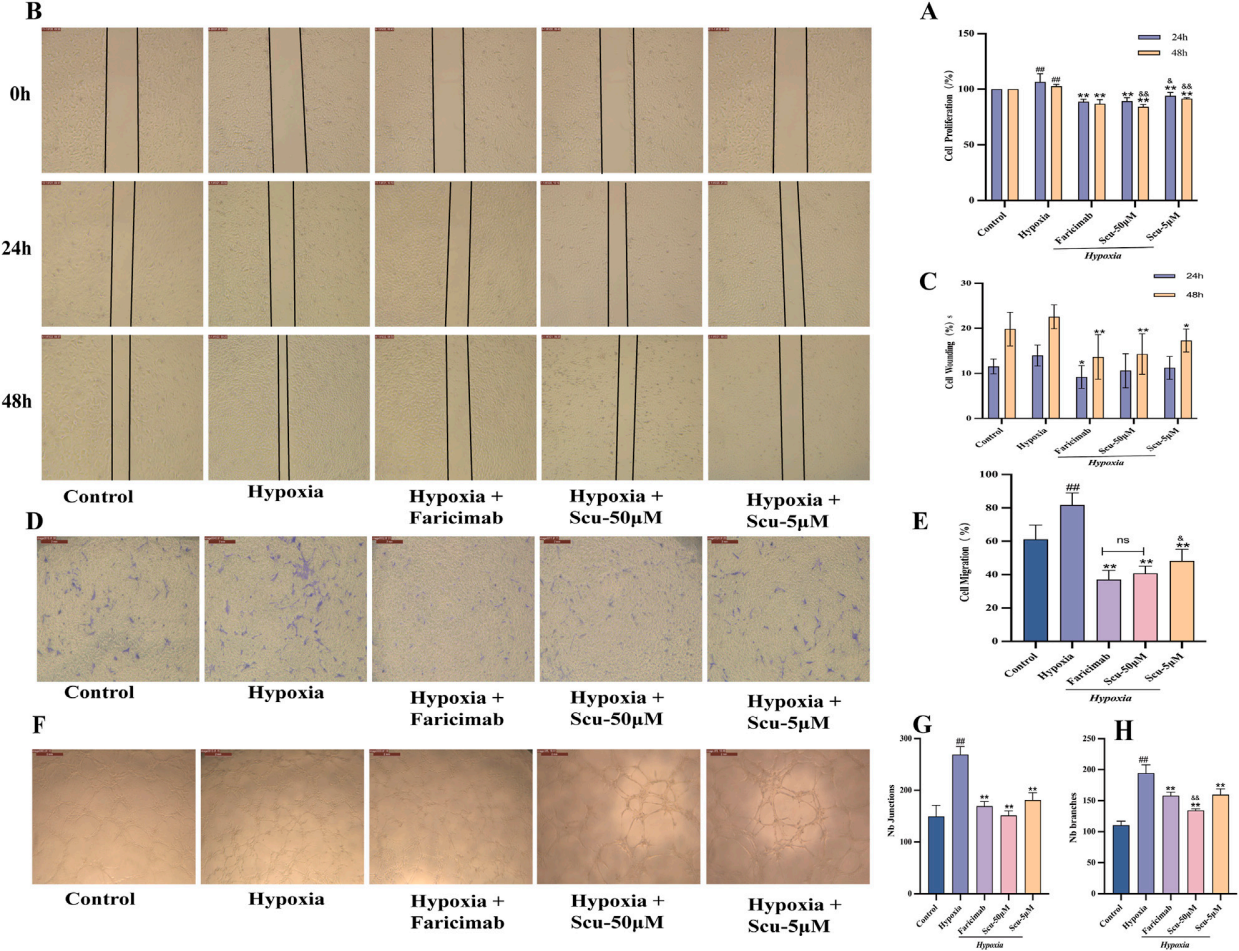
SCU and Faricimab attenuates hypoxia-induced proliferation, migration, and tube formation in rRMECs. **(A)** Proliferation rate as determined by CCK-8 assay. **(B)** Representative images of wound healing assay. **(C)** Quantitative analysis of migration area in scratch assay. **(D)** Transwell migration assay images. **(E)** Quantification of migrated cells (cell count per field). **(F)** Tube formation on Matrigel matrix. **(G)** Quantification of junctions. **(H)** Quantification of tube branches (nodes). Hypoxia group: CoCl_2_-induced model; Hypoxia + Faricimab group: 400 ng/mL. ^#^ *P* < 0.05, ^##^ *P* < 0.01 vs. Control group; ^*^ *P* < 0.05, ^**^ *P* < 0.01 vs. Hypoxia group; ^&^ *P* < 0.05, ^&&^ *P* < 0.01 vs. Hypoxia + Faricimab group; ns, no significance.

The CCK-8 assay results at both 24 h and 48 h demonstrated consistent trends (Figure 3A). Hypoxia significantly promoted rRMEC proliferation compared to the control group (*P* < 0.01). After 24 h of treatment, both SCU at 50 μmol/L and Faricimab at 400 ng/mL markedly inhibited hypoxia-induced proliferation (*P* < 0.01), with comparable effects and superior efficacy to the SCU-5 μmol/L group (*P* < 0.01). At 48 h, SCU-50 μmol/L exhibited a stronger inhibitory effect than Faricimab (*P* < 0.01).

### 3.4 SCU attenuates hypoxia-induced migration of rRMECs

Both scratch and Transwell assays were used to evaluate cell migration, and the results showed similar trends. The scratch assay (Figures 3B,C) demonstrated that hypoxia significantly increased rRMEC migration compared to the control group. Treatment with SCU (5 or 50 μmol/L) and Faricimab (400 ng/mL) for 24 h and 48 h significantly reduced migration area, with Faricimab showing slightly greater efficacy than SCU-50 μmol/L (*P* > 0.05). Both groups were more effective than SCU-5 μmol/L.

Transwell assay results (Figures 3D,E) further confirmed these findings. All three treatment groups (Faricimab, SCU-50 μmol/L, and SCU-5 μmol/L) significantly reduced the number of migrated cells under hypoxic conditions (*P* < 0.01). SCU-50 μmol/L and Faricimab showed comparable efficacy, both outperforming SCU-5 μmol/L.

### 3.5 SCU suppresses tube formation of rRMECs under hypoxic conditions

Tube formation ability was assessed by counting the number of junctions and branches. As shown in Figures 3F–H, hypoxia significantly enhanced the tube formation ability of rRMECs (*P* < 0.01). Treatment with SCU (5 or 50 μmol/L) and Faricimab (400 ng/mL) significantly inhibited tube formation under hypoxia (*P* < 0.01), as evidenced by reduced junction and branch numbers. The SCU-50 μmol/L group showed slightly better inhibition than both Faricimab and SCU-5 μmol/L.

### 3.6 Network pharmacology reveals potential multi-target pathways of SCU

#### 3.6.1 Identification of SCU–RNV intersection targets and construction of the PPI network

**FIGURE 4:**
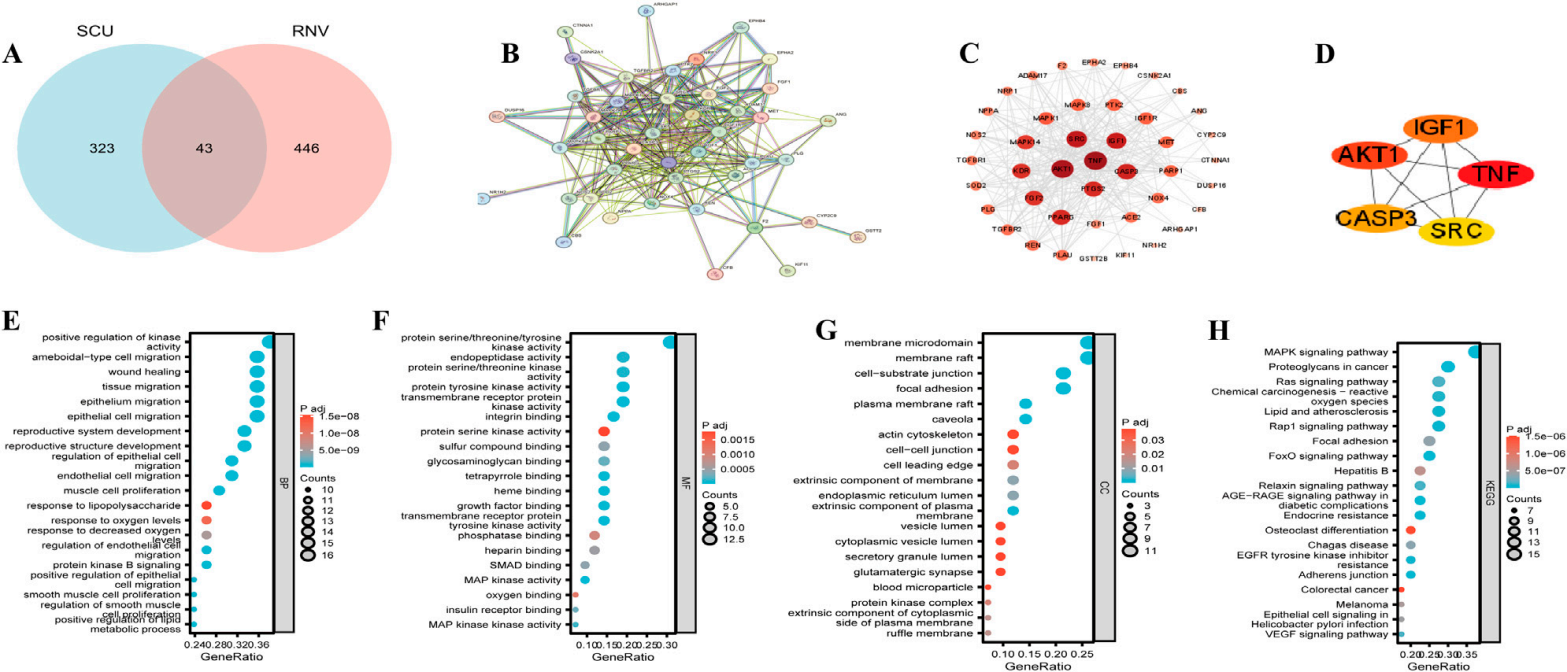
Network pharmacology analysis of SCU targeting the VEGF/Ang/Tie2 pathway. **(A)** Potential targets of SCU in the treatment of RNV; **(B)** PPI network of the intersected targets; **(C)** Visualization of the PPI network using Cytoscape; **(D)** Top five hub genes identified by CytoHubba; **(E)** GO enrichment analysis for BP; **(F)** Molecular function (MF); **(G)** Cellular component (CC); **(H)** Top 20 enriched pathways from GO and KEGG analyses.

A total of 366 potential targets of SCU were predicted using the PharmMapper and Swiss Target Prediction databases. In parallel, 489 RNV-related targets were retrieved from the GeneCards database. The intersection of these datasets yielded 43 putative targets of SCU in the treatment of RNV (Figure 4A). These targets were input into the STRING database to construct a protein-protein interaction (PPI) network (Figure 4B), which revealed significant interactions among them. The network was visualized using Cytoscape 3.9.1 (Figure 4C), where larger and darker nodes indicated greater relevance to RNV. The top five hub genes—TNF, AKT1, SRC, IGF1, and CASP3—were identified using the MCC algorithm in the cytoHubba plugin (Figure 4D).

#### 3.6.2 GO and KEGG enrichment analysis

GO and KEGG enrichment analyses were conducted for the 43 intersection targets. In the GO biological process (BP) category (Figure 4E), the enriched terms included endothelial cell migration, response to oxygen levels, and response to hypoxia. In the molecular function (MF) category (Figure 4F), enriched terms involved protein serine/threonine/tyrosine kinase activity, endopeptidase activity, and protein tyrosine kinase activity. In the cellular component (CC) category (Figure 4G), targets were enriched in membrane rafts, cytoplasmic vesicle lumen, and secretory granule lumen. KEGG pathway analysis (Figure 4H) indicated significant enrichment in the MAPK, Ras, and VEGF signaling pathways, among which the VEGF pathway showed the most direct relevance to RNV. These findings suggest that SCU may exert anti-RNV effects via multiple angiogenesis-related signaling pathways.

#### 3.6.3 Molecular docking confirms SCU affinity to VEGF/Ang/Tie2 targets

**FIGURE 5:**
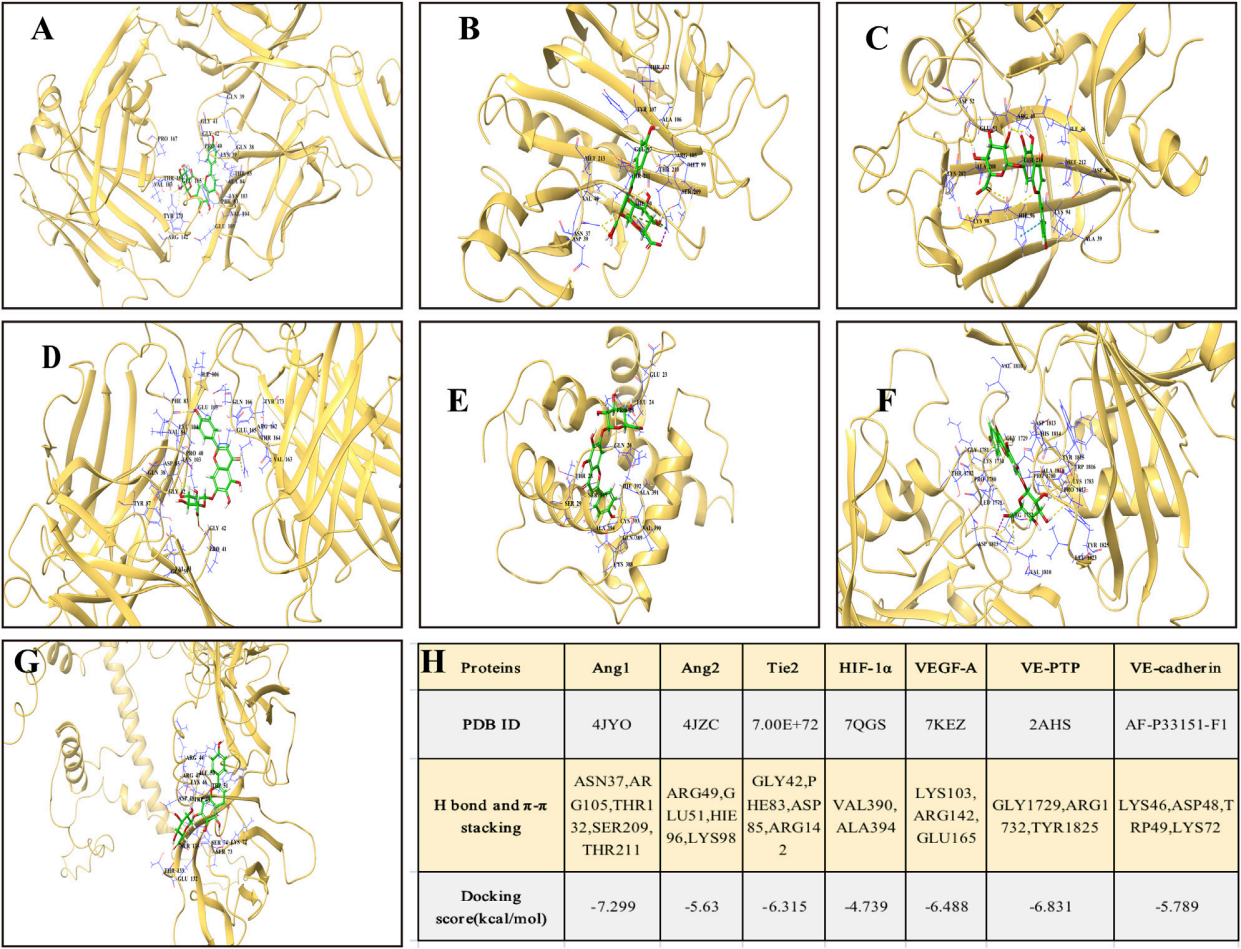
Molecular docking analysis of SCU with key targets in the VEGF/Ang/Tie2 signaling pathway. Three-dimensional structures of SCU docked with **(A)** Ang1; **(B)** Ang2; **(C)** Tie2 receptor; **(D)** HIF-1α; **(E)** VEGF-A, **(F)** VE-PTP; **(G)** VE-cadherin; **(H)** Molecular docking analysis of SCU with key targets in VEGF/Ang/Tie2 signaling pathway.

To validate the predictions from network pharmacology, molecular docking was performed between SCU and key proteins in the VEGF/Ang/Tie2 pathway, including Ang1, Ang2, Tie2, HIF-1α, VEGF-A, VE-PTP, and VE-cadherin. The results (Figure 5) showed that SCU exhibited favorable binding affinities with these proteins. The binding energies for Ang1 (−7.299 kcal/mol), VEGF-A (−6.488 kcal/mol), Tie2 (−6.315 kcal/mol), and VE-PTP (−6.831 kcal/mol) were especially low, indicating stable interactions. These findings suggest that SCU may exert dual regulatory effects on the VEGF/Ang/Tie2 pathway by directly targeting multiple key proteins, thereby suppressing pathological angiogenesis while promoting vascular stability.

### 3.7 SCU regulates VEGF/Ang/Tie2-related protein expression in rRMECs

**FIGURE 6:**
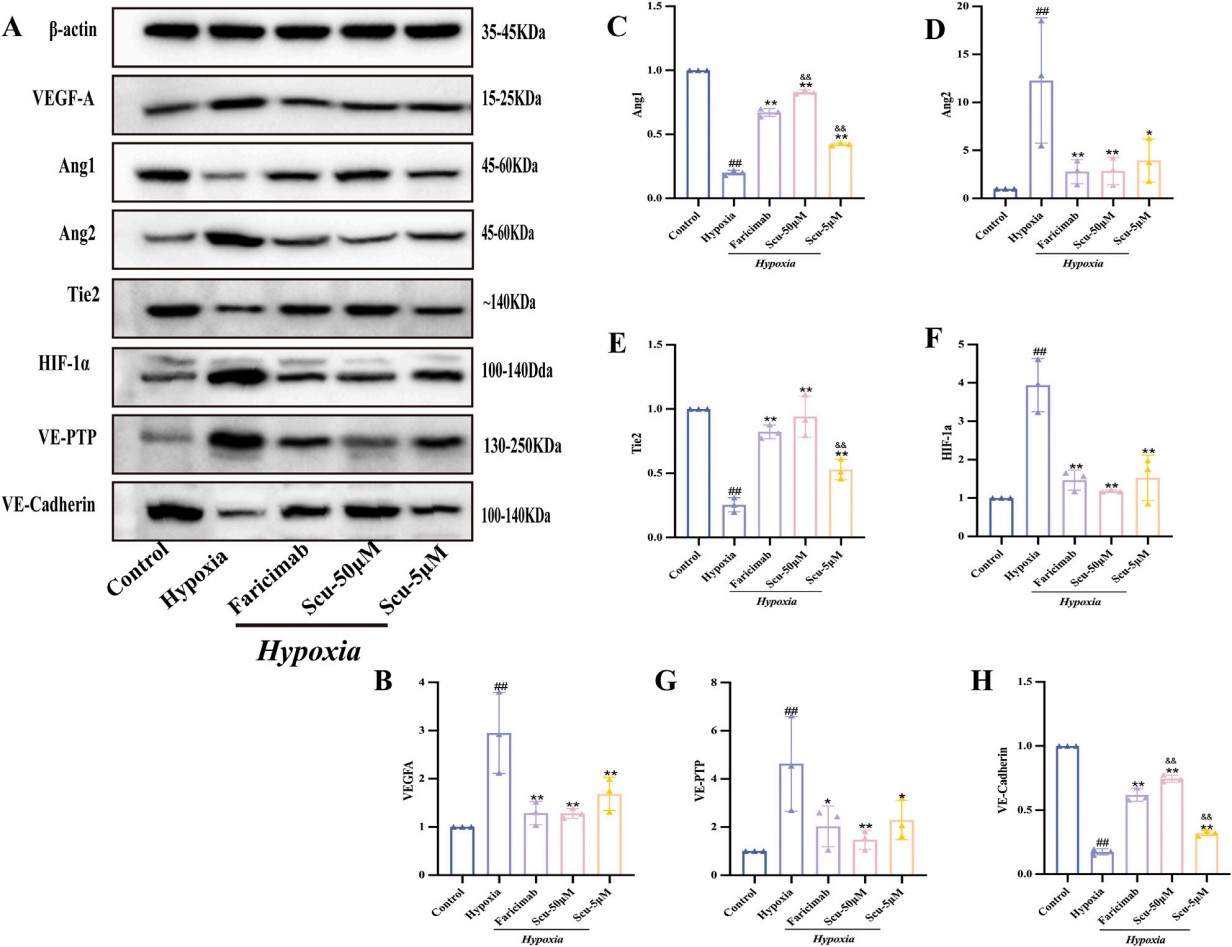
Effects of SCU on the protein expression of VEGF/Ang/Tie2 pathway-related targets in rRMECs. **(A)** Representative Western blot bands. **(B–H)** Quantification of protein expression levels of VEGF-A, Ang1, Ang2, Tie2, HIF-1α, VE-PTP, and VE-Cadherin. ^#^ *P* < 0.05, ^##^ *P* < 0.01 vs. Control group; ^*^ *P* < 0.05, ^**^ *P* < 0.01 vs. Hypoxia group; ^&^ *P* < 0.05, ^&&^ *P* < 0.01 vs. Hypoxia + Faricimab group.

To verify the results of molecular docking, Western-blot analysis was conducted to examine the expression of key proteins in the VEGF/Ang/Tie2 signaling pathway. As shown in Figure 6, compared with the control group, hypoxia significantly downregulated the protein expression levels of Ang1, Tie2, and VE-cadherin (*P* < 0.01), while markedly upregulating the levels of Ang2, HIF-1α, VEGF-A, and VE-PTP (*P* < 0.01), confirming successful model induction and pathological activation of angiogenic signaling. Following 24 h of treatment, both the SCU (5 and 50 μmol/L) and Faricimab (400 ng/mL) groups demonstrated suppression of Ang2, HIF-1α, VEGF-A, and VE-PTP protein expression (*P* < 0.05) compared to the hypoxia group, while concurrently upregulating Ang1, Tie2, and VE-cadherin (*P* < 0.01), in a dose-dependent manner. Notably, the SCU-50 μmol/L group exhibited the most pronounced regulatory effect, and its enhancement of Ang1, Tie2, and VE-cadherin expression exceeded that of Faricimab, with a statistically significant difference for Ang1 and VE-cadherin (*P* < 0.01), although not for Tie2 (*P* > 0.05).

### 3.8 SCU modulates mRNA expression of VEGF/Ang/Tie2 pathway components

**FIGURE 7:**
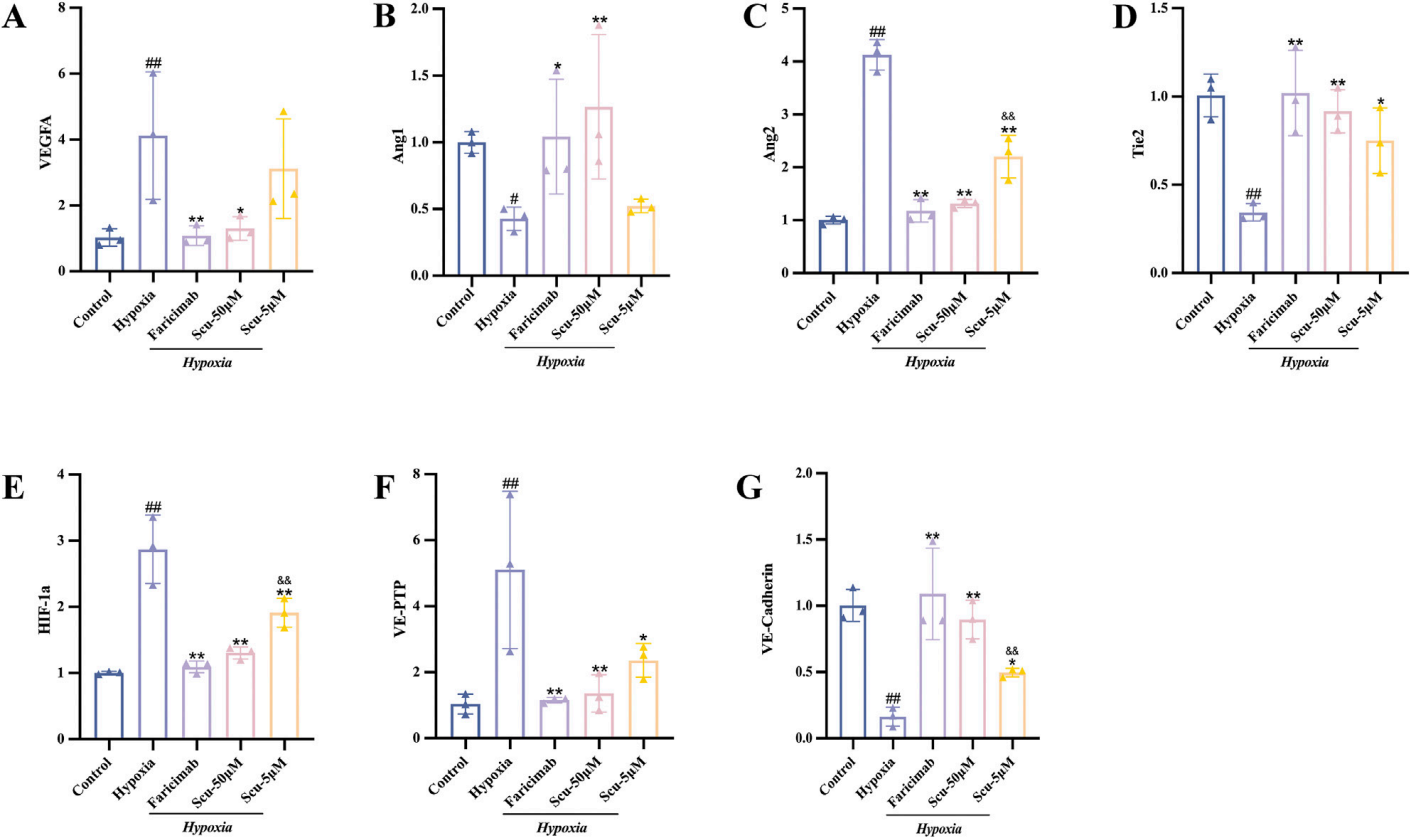
Effects of SCU on mRNA expression of VEGF/Ang/Tie2 pathway-related targets in rRMECs. **(A–G)** Relative mRNA expression levels of VEGF-A, Ang1, Ang2, Tie2, HIF-1α, VE-PTP, and VE-Cadherin measured by qRT-PCR. ^#^ *P* < 0.05, ^##^ *P* < 0.01 vs. Control group; ^*^ *P* < 0.05, ^**^ *P* < 0.01 vs. Hypoxia group; ^&^ *P* < 0.05, ^&&^ *P* < 0.01 vs. Hypoxia + Faricimab group.

qRT-PCR analysis was performed to further validate the transcriptional regulation of VEGF/Ang/Tie2 pathway-related genes by SCU. As shown in Figure 7, hypoxia reduced the mRNA levels of Ang1, Tie2, and VE-cadherin while elevating Ang2, HIF-1α, VEGF-A, and VE-PTP expression compared to the control group (*P* < 0.05), consistent with protein-level changes. After intervention, both SCU and Faricimab reversed hypoxia-induced mRNA expression changes. Specifically, SCU at 5 and 50 μmol/L and Faricimab significantly downregulated Ang2, HIF-1α, VEGF-A, and VE-PTP mRNA levels (*P* < 0.05) and upregulated Ang1, Tie2, and VE-cadherin (*P* < 0.05).

## 4 Discussion

Retinal neovascularization (RNV) is a complex multistep process involving basement membrane degradation, endothelial cell (ECs) activation, proliferation, migration, and tube formation (Ren et al., 2020). Hypoxia critically contributes to the initiation of RNV(Alizadeh et al., 2018), primarily by enhancing the expression of hypoxia-inducible factor-1α (HIF-1α), which subsequently promotes VEGF-mediated angiogenesis (Zhao et al., 2022). VEGF-A, recognized as the predominant isoform within the VEGF family (Li et al., 2020; Khanani et al., 2021), stimulates ECs proliferation and migration (Gunaratnam and Bonventre, 2009), remodeling of the extracellular matrix (Claesson-Welsh and Welsh, 2013), and increased vascular permeability under hypoxic conditions and augments vascular permeability particularly under hypoxic environments (Xu et al., 2018). However, anti-VEGF monotherapy often leads to vascular fragility and does not fully stabilize neovasculature (Chaudhary et al., 2024), highlighting the need for dual-pathway strategies.

**FIGURE 8:**
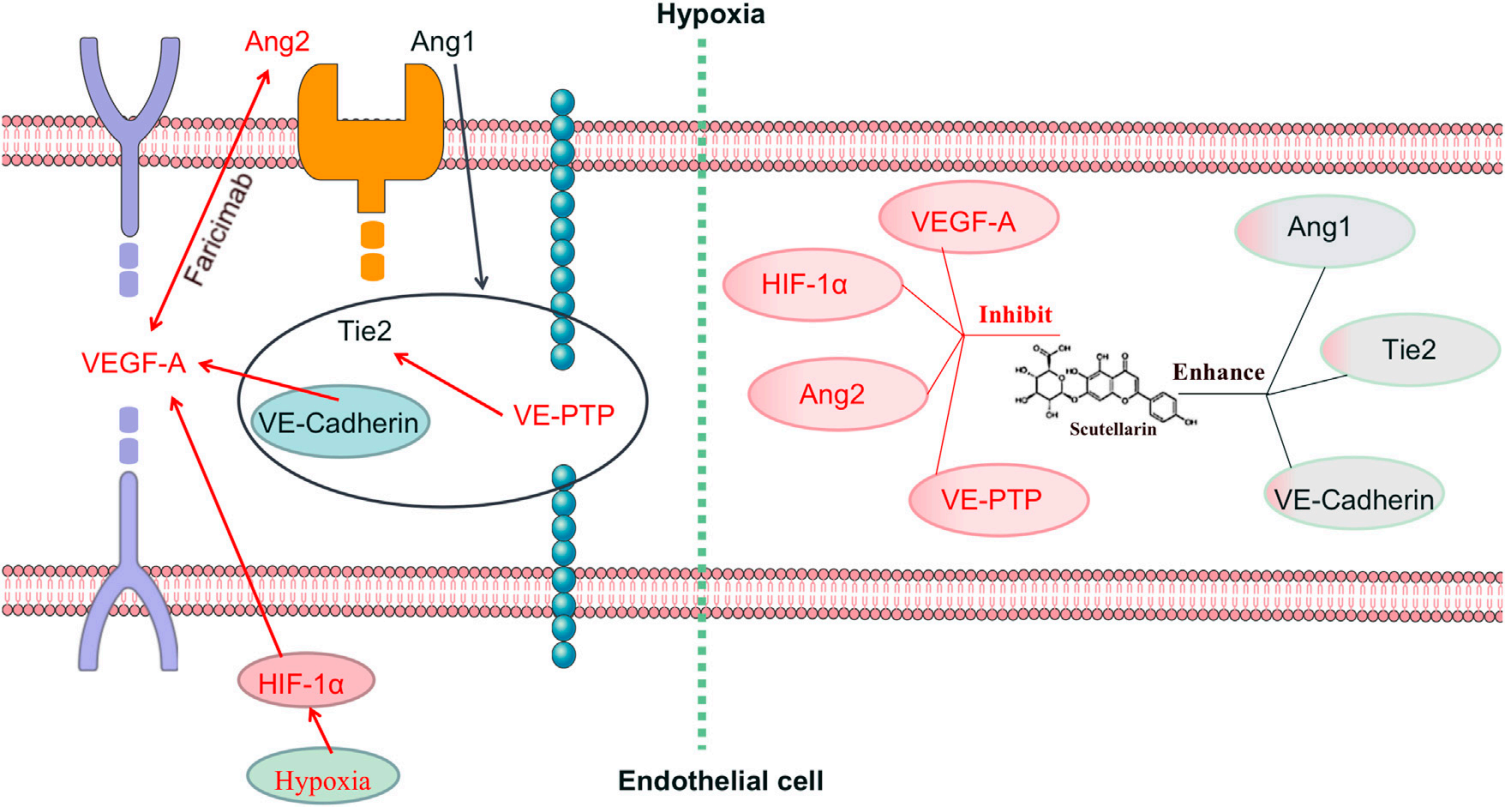
Schematic illustration of the bidirectional regulatory effects of Scutellarin on hypoxia-induced retinal neovascularization. Red indicates pro-angiogenic or destabilizing effects; black indicates vascular-stabilizing effects.

The Angiopoietin/Tie2 pathway serves as a key regulatory mechanism in preserving vascular integrity and promoting vessel maturation (Whitehead et al., 2019). Tie2, predominantly localized in endothelial cells, orchestrates vascular remodeling processes and the maintenance of vascular homeostasis (Zhang et al., 2021). Its ligand Ang1 promotes EC junctions and neovascular stability by activating Tie2, counteracting VEGF-driven angiogenesis (Akwii and Mikelis, 2021). Ang1 also facilitates the formation of Tie2/VE-cadherin/VE-PTP complexes, reducing vascular permeability (Saharinen et al., 2008). In contrast, Ang2, which is upregulated under hypoxic conditions, antagonizes Ang1-mediated Tie2 signaling and destabilizes the endothelium, thereby synergizing with VEGF to promote pathological angiogenesis (Saharinen et al., 2017; Jiang et al., 2018; Akwii et al., 2019). VE-PTP, a key negative regulator of Tie2, is also elevated in hypoxia and contributes to Tie2 deactivation (Akwii and Mikelis, 2021) and vascular destabilization (Souma et al., 2018). Additionally, phosphorylated VE-cadherin mediates EC transition from a quiescent to an angiogenic state via the VEGF pathway (Mirando et al., 2019). Therefore, balancing VEGF and Ang/Tie2 signaling—particularly the Ang1/Ang2-Tie2 axis—is crucial for achieving dual modulation of angiogenesis and vascular stabilization in RNV (Figure 8).

In this study, we explored the regulatory effect of SCU, a flavonoid derived from *E. breviscapus*, widely used in Traditional Chinese Medicine for treating blood stasis-related ocular disorders. SCU markedly inhibited critical cellular behaviors associated with RNV, such as hypoxia-stimulated proliferation, migratory activity, and capillary-like tube formation in rRMECs. Network pharmacology and molecular docking analyses suggested that SCU targets multiple signaling pathways, with the VEGF/Ang/Tie2 axis being central. SCU showed strong binding affinities to VEGF-A, Ang2, Tie2, and VE-PTP. Western blot and qRT-PCR confirmed that SCU downregulated VEGF-A, Ang2, HIF-1α, and VE-PTP, while upregulating Ang1, Tie2, and VE-cadherin in a dose-dependent manner. These findings support a bidirectional regulatory mechanism: inhibiting pathological angiogenesis while enhancing vascular stabilization.

Interestingly, SCU shares partial mechanistic similarity with Faricimab, an approved bispecific antibody for neovascular retinal diseases. Which acts extracellularly by neutralizing VEGF-A and Ang2, yet this blockade triggers intracellular feedback signaling cascades that reshape gene expression. Specifically, VEGF-A removal diminishes VEGFR2 activation, thereby downregulating the PI3K/Akt/HIF-1α axis, which is known to stabilize HIF-1α and enhance VEGF-A transcription under hypoxic conditions (Avrutsky et al., 2023). Simultaneously, Ang2 blockade releases its antagonism on Tie2 by preventing VE-PTP recruitment, thereby enhancing Ang1 mediated Tie2 phosphorylation (Khan et al., 2020). This restored Tie2 activity stabilizes endothelial junctions through VE-Cadherin membrane localization and suppresses VE-PTP transcription (Hellström et al., 2007). These receptor-level modulations lead to transcriptional feedback loops, explaining the downregulation of VEGF-A, Ang2, VE-PTP, and HIF-1α, and upregulation of Ang1, Tie2, and VE-Cadherin at both the mRNA and protein levels. Thus, although Faricimab acts extracellularly, its dual-ligand blockade reshapes intracellular signaling and transcription networks through receptor deactivation and signal-dependent feedback mechanisms. However, unlike Faricimab, SCU not only inhibits upstream angiogenic stimuli but also upregulates Tie2 and VE-cadherin expression, suggesting direct modulation of vascular integrity pathways via intracellular targets rather than ligand sequestration alone.

This study is the first to systematically demonstrate that SCU exerts anti-RNV effects via bidirectional regulation of the VEGF/Ang/Tie2 signaling pathway. This dual action not only addresses the limitations of current anti-VEGF monotherapies but also underscores SCU’s synergistic potential in inhibiting angiogenesis and promoting vascular stability. These findings offer a novel direction for the development of multi-targeted herbal interventions for retinal vascular diseases.

Notably, although SCU treatment increased Tie2 expression at both mRNA and protein levels, this alone does not confirm pathway direct activation, as Tie2 signaling typically requires phosphorylation events that were not evaluated in this study (Nguyen et al., 2020). Supporting this possibility, a previous study from our group using Qideng Mingmu Capsules demonstrated elevated p-Akt levels along with Tie2 upregulation, indirectly suggesting downstream activation of the Ang/Tie2 pathway (Liu et al., 2025). Although Tie2 phosphorylation was not directly assessed in the present study, these earlier findings lend indirect support to the involvement of the Ang/Tie2 axis in SCU-mediated vascular protection. The lack of p-Tie2 detection represents a mechanistic limitation. Future work will include Western-blot to assess Tie2 phosphorylation and downstream signaling molecules such as Akt and FOXO1, thereby providing direct evidence of pathway activation.

However, this study still has several limitations. First, the findings are based on *in vitro* models, lacking *in vivo* confirmation. Second, the use of CoCl_2_ to induce hypoxia may not fully replicate the complex dynamic oxygen fluctuations observed in physiological conditions. To address this, future studies will incorporate more physiologically relevant models, such as hypoxia chambers for precisely controlled oxygen conditions *in vitro*, and oxygen-induced retinopathy mouse models to assess SCU’s therapeutic efficacy *in vivo*. Third, although molecular docking suggested strong binding of SCU to several angiogenic proteins, no binding assays (e.g., SPR, ITC, or saturation/inhibition kinetics) were conducted to confirm these interactions. This limits the interpretation of SCU’s direct binding effects and receptor activity modulation. Further investigations including animal studies, transcriptomic profiling, and preclinical pharmacological evaluations are essential to fully elucidate SCU’s mechanisms and therapeutic feasibility.

## 5 Conclusion

In conclusion, this study demonstrate that SCU exerts a dual regulatory effect on the VEGF/Ang/Tie2 pathway, simultaneously inhibiting pathological angiogenesis and enhancing vascular stability under hypoxic conditions. By integrating experimental pharmacology with computational approaches, including network pharmacology, molecular docking, and *in vitro* assays, we identified SCU as a promising multi-target natural compound for retinal neovascularization. These findings provide a pharmacological basis for SCU’s potential therapeutic application in retinal vascular diseases. Future *in vivo* studies and clinical investigations are warranted to validate these results and explore the translational potential of SCU in ophthalmic therapy.

## Data Availability

The data presented in the study are deposited in the Figshare repository, available at doi:10.6084/m9.figshare.30198229.

## Abbreviations

SCU, Scutellarin; RNV, Retinal neovascularization; VEGF, Vascular endothelial growth factor; Ang, Angiopoietin; VE-PTP, Vascular endothelial-protein tyrosine phosphatase; VE-cadherin, Vascular endothelial cadherin; nAMD, neovascular age-related macular degeneration; DME, Diabetic macular edema; rRMECs, Rat retinal microvascular endothelial cells; CoCl_2_, Cobalt chloride; ECM, Endothelial Cell Medium; FBS, Fetal bovine serum; PBS, Phosphate-buffered saline; HRP, Horseradish peroxidase; qRT-PCR, Quantitative real-time polymerase chain reaction; CDUTCM, Chengdu University of Traditional Chinese Medicine; TCM, Traditional Chinese Medicine; FN, Fibronectin; vWF, von Willebrand factor antibody; ANOVA, One-way analysis of variance; LSD, least significant difference; GO, Gene Ontology; PPI, Protein-protein interaction; KEGG, Kyoto Encyclopedia of Genes and Genomes; BP, biological process; MF, Molecular function; CC, Cellular component.
